# Foliar Application of 24-Epibrassinolide Improves Growth, Ascorbate-Glutathione Cycle, and Glyoxalase System in Brown Mustard (*Brassica juncea* (L.) Czern.) under Cadmium Toxicity

**DOI:** 10.3390/plants9111487

**Published:** 2020-11-04

**Authors:** Pravej Alam, Sukhmeen Kaur Kohli, Thamer Al Balawi, Fahad H. Altalayan, Prawez Alam, Muhammad Ashraf, Renu Bhardwaj, Parvaiz Ahmad

**Affiliations:** 1 Department of Biology, College of Science and Humanities, Prince Sattam bin Abdulaziz University, Al-Kharj 11942, Saudi Arabia; thameralbalawi@gmail.com (T.A.B.); fahadhaltalayan@gmail.com (F.H.A.); 2 Plant Stress Physiology Lab, Department of Botanical and Environment Sciences, Guru Nanak Dev University, Amritsar 43005, India; sukhmeenkohli@gmail.com (S.K.K.); renubhardwaj82@gmail.com (R.B.); 3 Department of Pharmacognosy, College of Pharmacy, Prince Sattam bin Abdulaziz University, Al-Kharj 11942, Saudi Arabia; p.alam@psau.edu.sa; 4 Department of Botany, Faculty of Science, University of Agriculture Faisalabad, Faisalabad 38000, Pakistan; ashrafbot@yahoo.com; 5 Botany and Microbiology Department, College of Science, King Saud University, Riyadh 11451, Saudi Arabia; 6 Department of Botany, S.P. College Srinagar, Jammu and Kashmir, Srinagar 190001, India

**Keywords:** cadmium, 24-Epibrassinolide, antioxidant enzymes, osmoprotectants, malondialdehyde, transpiration rate

## Abstract

Cadmium (Cd) metal toxicity is a crucial ecological matter that requires immediate efforts to mitigate it. *Brassica juncea* plants were exposed to Cd (0 and 200 µM as CdSO_4_) and foliar application of 24-Epibrassinolide (EBR) (0, 10^−7^ and 10^−5^ M). The toxic effect of Cd was evident in terms of declined growth and biomass yield, lowered levels of pigment content and chlorophyll fluorescence, and reduction in gas exchange attributes. The levels of proline and glycinebetaine increased in response to Cd treatment. There was an imperative rise in the contents of H_2_O_2_ and malondialdehyde as well as electrolyte leakage in the Cd-stressed plants. With the application of EBR, there was a significant replenishment in growth attributes and photosynthetic efficacy. The contents of ROS (reactive oxygen species) and malondialdehyde as well as electrolyte leakage were reduced by the hormone supplementation. Enhancement in the contents of glutathione and ascorbic acid, and the activities of enzymes of the antioxidative defense system and glyoxalase system was recorded in response to Cd as well as hormone treatment. The in situ levels of Cd in roots and shoot were augmented in response to Cd treatment, but were found to be lowered by the EBR application.

## 1. Introduction

The impending intimidation to the environment and human wellbeing is ascribed to the presence of immensely high levels of heavy metals in the surroundings [1,2]. In response to a stress caused by the intake of the heavy metals (e.g., lead, chromium, cadmium), plants undergo alterations in gene expression and metabolic processes. These heavy metals are not required by the plants, and above an optimal limit, they are toxic to plants [3,4]. Cadmium (Cd) is included amongst the most toxic heavy metals present within the soil and water bodies [5,6]. Cadmium uptake and sequestration in plants and their consequent movement into the food chain and ecosystem have awakened huge civic apprehensions worldwide. Cadmium entering into the environment by human activities, accumulates in the water resources and earth, which eventually results in bio-magnification in the agricultural produce, thereby negatively impacting the crop productivity [7,8,9]. A wide array of experimental evidence reveals the noxious influence of Cd on plants [5,10,11]. Surfeit levels of Cd harmfully affect growth and subsequently lead to toxicities at functional and cellular levels [7]. Cadmium toxicity had hazardous impact on growth and sustainability of plants by down-regulation of the chlorophyll production and variations in ultra-structure of plant cells [5,12]. Plants under Cd stress generate high amounts of reactive oxygen species (ROS) as well as malondialdehyde (MDA) [12]. Furthermore, raised synthesis of ROS may also influence the cells and cause damage thereto, eventually resulting in necrosis of plant species [13]. Hence, there is a critical necessity to reduce Cd sequestration in plants using efficient and environment-friendly measures.

To evade the toxic effect of metal toxicity, plants employ a myriad of strategies by which the toxic ions in the plant system are effluxed, localized in the roots, or accumulate in other plant parts [14]. These strategies are generally categorized into avoidance and detoxification approaches [15]. One of the few avoidance strategies involves enhanced activities of antioxidative defense enzymes and levels of non-enzymatic antioxidants [16]. More lately, employment of exogenous plant growth regulators to provide a defense to plants against oxidative burst has gained a lot of attention [17,18,19]. A wide array of phytohormones viz. auxins, gibberellins, brassinosteroids, ethylene, salicylic acid, jasmonic acid, and many others have been studied for their optimistic potential to enhance growth and tolerance of plants exposed to heavy metal toxicity [20,21,22,23,24,25,26].

One of the chief classes of phytohormones is brassinosteroids (BRs); they are phytosteroidal in nature and modulate growth and development activities including cell maturation, seed germination, vascular differentiation, photosynthesis and development of reproductive system, etc. [27]. Despite their essential involvement in growth regulation under normal growth conditions, they also provide tolerance to plants against various environmental stresses [28,29]. These phytosteroids have been widely reported to alleviate cadmium toxicity by replenishing photosynthetic attributes [30] such as protection of photosynthetic apparatus and electron transport chain [31], elevation in activities of antioxidative defense components, and also the transcript levels of secondary metabolites [31,32].

Several members of the Brassicaceae family including *Brassica napus*, *Brassica oleracea* and *B. juncea* have been extensively reported as accumulators of heavy metals [33]. *Brassica juncea* (Brown mustard) is widely cultivated in different parts of the globe. Additionally, it can accumulate heavy metals and thus was employed as a model plant in the present study. Cadmium unfavorably influences the physio-biochemical features of *B. juncea* plants, which in due course results in diminished crop productivity [34]. The hypothesis, thus intended to be tested, was that up to what extent exogenous application of EBR could mitigate plant growth, and regulate photosynthetic attributes, antioxidant defense system and osmoregulating ability of *B. juncea* plants under Cd toxicity.

## 2. Results

### 2.1. Improvement in Mustard Growth by 24-EBR Application

**Figure 1:**
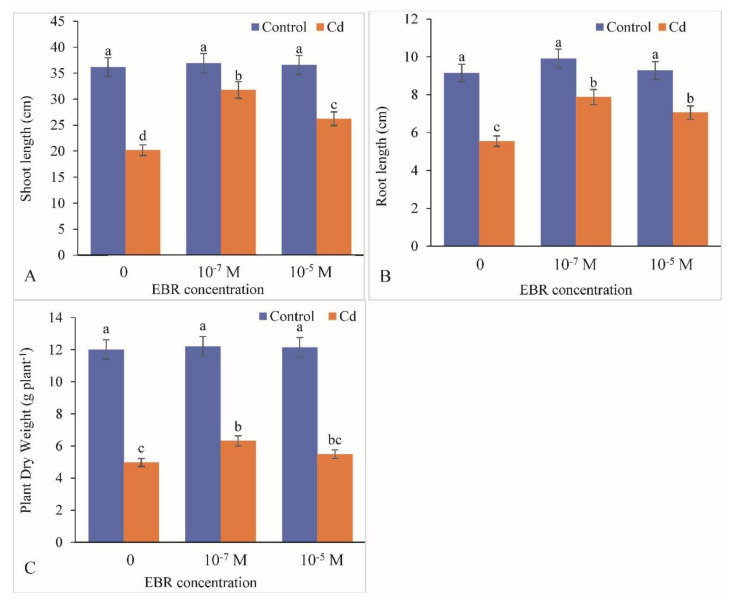
Foliar application of 24-Epibrassinolide (EBR) enhanced length of (**A**) shoot, (**B**) root and (**C**) plant dry weight in *B. juncea* under Cd (200 µM) stress. Values with different letters above the bars are significantly different at each measured time point (*p* < 0.05) (Mean ± S.E.).

The effects of foliar application of 24-EBR on mustard growth in terms of biomass production and shoot length are presented in Figure 1. Under Cd stress, the shoot length decreased by 44.18% compared to the control. Foliar supplementation of 24-EBR enhanced shoot length by 57.30% and 29.86% at 10^−7^ and 10^−5^ M EBR treatments, respectively, compared with those of plants treated with Cd only (Figure 1A). A similar decline of 39.34% was observed in root length with respect to that in the control. The root length was replenished significantly by 41.98% and 27.20% when supplied with 10^−7^ and 10^−5^ M EBR treatments with reference to that in plants supplied with Cd only (Figure 1B). The dry weight of plants was recorded to be lowered by 58.56% under Cd stress. In response to foliar application of 24-EBR (10^−7^ and 10^−5^ M), the dry weights of plants increased by 26.90% and 10.24%, respectively, in comparison with the controls (Figure 1C).

### 2.2. Enhancement in Chlorophyll and Carotenoid levels by 24-EBR Supplementation

**Figure 2:**
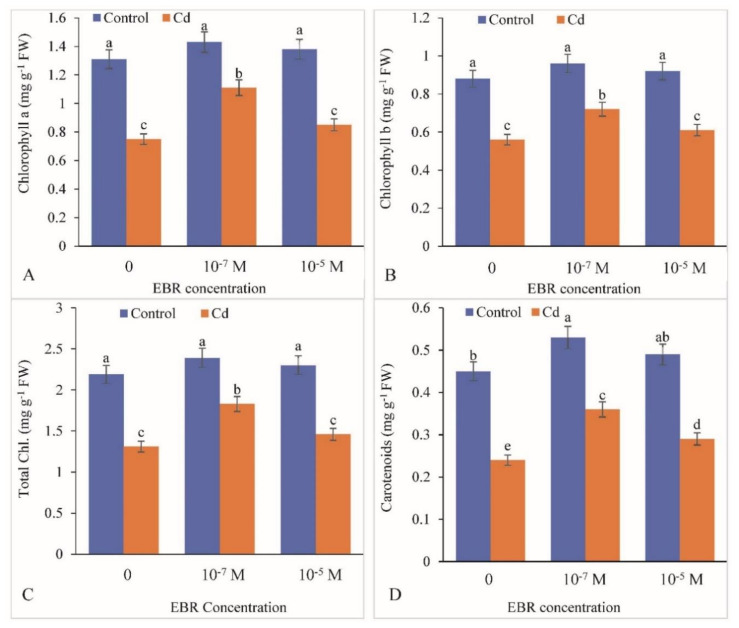
24-Epibrassinolide restores the (**A**) chlorophyll a, (**B**) chlorophyll b, (**C**) total chlorophyll and (**D**) carotenoid content in the leaves of *B. juncea* under Cd (200 µM) stress. Values with different letters above the bars are significantly different at each measured time point (*p* < 0.05) (Mean ± S.E.).

Chlorophyll a and b contents were observed to be significantly reduced by 42.74% and 36.36%, respectively, in response to Cd stress when compared with the controls. These attributes were notably enhanced by foliar application of 24-EBR (Figure 2A–D). Chlorophyll a and b concentrations showed 48% and 13.33% rise at 10^−7^ and 10^−5^ M of EBR, respectively, in comparison with those in plants fed with Cd only (Figure 2A,B). Similarly, total chlorophyll and carotenoid levels decreased by 48.40% and 46.66%, respectively, in Cd stress plants compared to the controls (Figure 2C,D). However, there was a rise of 39.69% and 11.45% in response to 10^−7^ and 10^−5^ M of 24-EBR application, respectively.

### 2.3. Variation in Photosynthetic Parameters (Chlorophyll Fluorescence)

**Figure 3:**
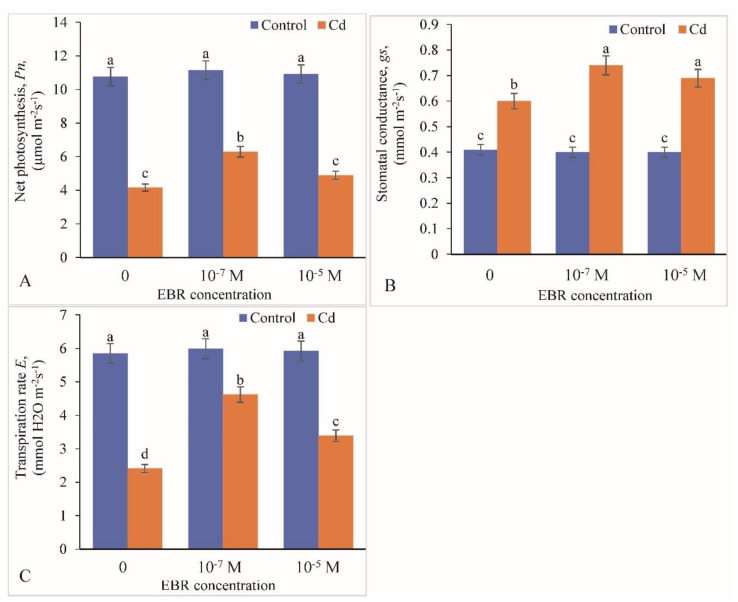
External supplementation of 24-Epibrassinolide maintains the (**A**) net photosynthesis, (**B**) stomatal conductance and (**C**) transpiration rate in the leaves of *B. juncea* under Cd (200 µM) stress. Values with different letters above the bars are significantly different at each measured time point (*p* < 0.05) (Mean ± S.E.).

Net photosynthesis rate and transpiration rate decreased by 61.37% and 58.80%, respectively; however, stomatal conductance increased by 46.34% in Cd stress plants compared with the controls (Figure 3A–C). A linear elevation in net photosynthetic rate, stomatal conductance and transpiration rate were recorded with increase in 24-EBR supply (Figure 3). The net photosynthesis rose by 51.20% and 17.54% in 10^−7^ and 10^−5^ M 24-EBR, respectively, compared with the controls. The enhancement in stomatal conductance (23.33% and 15%) and transpiration rate (91.70% and 40.66%) were observed in 10^−7^ and 10^−5^ M 24-EBR-supplemented plants, respectively, in comparison with the control plants (Figure 3A–C).

**Figure 4:**
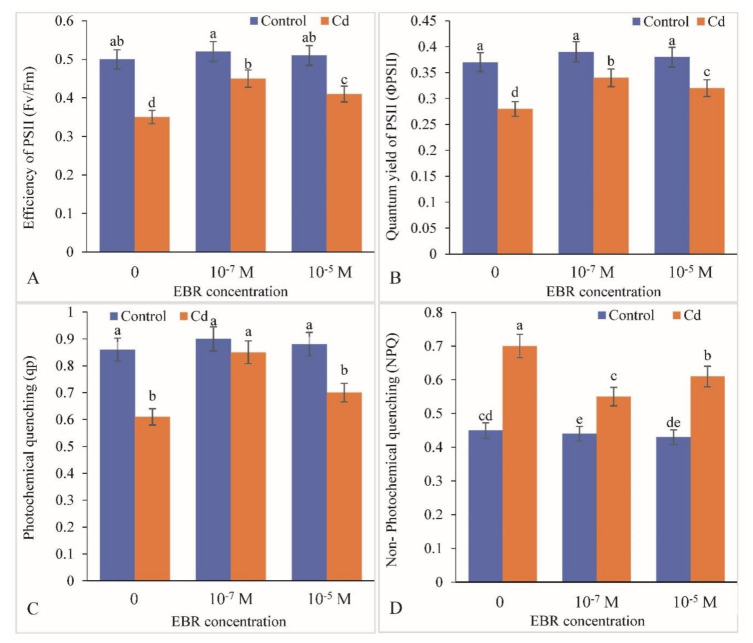
Supplementation of 24-Epibrassinolide boosts (**A**) Fv/Fm, (**B**) ΦPSII, (**C**) qp and (**D**) declines NPQ in the leaves of *B. juncea* under Cd (200 µM) stress. Values with different letters above the bars are significantly different at each measured time point (*p* < 0.05) (Mean ± S.E.).

The efficiency of photosystem II (PSII) was recorded to be decreased by 50.0% in Cd-stressed plants relative to the control. Exogenously supplied 24-EBR viz. 10^−7^ and 10^−5^ M resulted in up-regulation of the PSII efficiency by 84.0% and 32.0% respectively, with respect to that in the controls (plants treated with Cd only) (Figure 4A). A similar enhancement in quantum yield of PSII and photochemical quenching was observed by foliar application of 24-EBR. Quantum yield of PSII was augmented by 57.17% and 33.33% and photochemical quenching by 39.34% and 14.75%, respectively, in 10^−7^ and 10^−5^ M 24-EBR- supplemented plants when compared with Cd-alone-treated plants (Figure 4B,C). On the contrary, non-photochemical quenching was up-regulated in response to Cd stress by 55.55%, whereas 24-EBR treatment viz. 10^−7^ and 10^−5^ M resulted in lowering the same by 28.58% and 12.86%, respectively (Figure 4D).

### 2.4. RWC, Proline and Glycine Betaine

**Figure 5:**
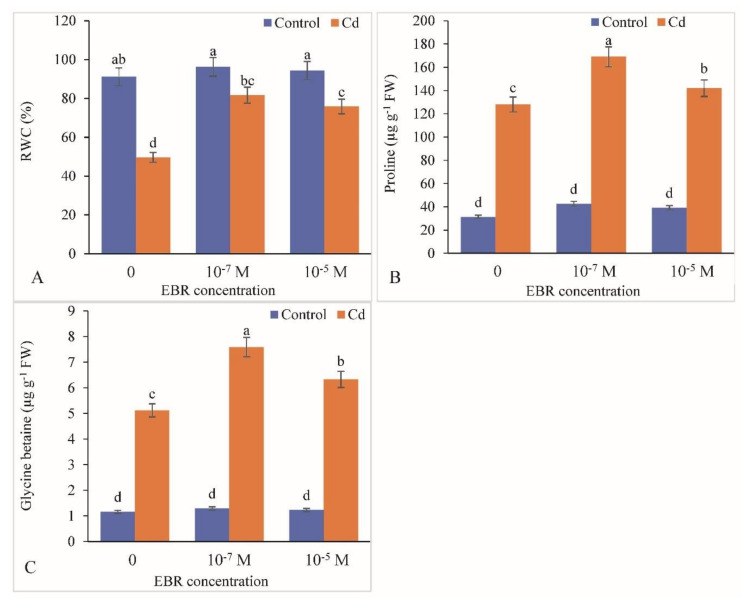
Application of 24-Epibrassinolide enhanced leaf (**A**) RWC, (**B**) proline content and (**C**) glycine betaine in *B. juncea* under Cd (200 µM) stress. Values with different letters above the bars are significantly different at each measured time point (*p* < 0.05) (Mean ± S.E.).

Under Cd stress, RWC decreased by 45.53% with respect to the control. Application of 24-EBR showed a rise in RWC by 64.55% and 52.77% in 10^−7^ and 10^−5^ M 24-EBR Cd-stressed plants (Figure 5A). Proline levels in leaf samples increased by 32.03% and 10.93% with 10^−7^ and 10^−5^ M 24-EBR treatments, respectively (Figure 5B). Similarly, GB concentration was significantly improved by 48.24% and 23.63% in 10^−7^ and 10^−5^ M 24-EBR-supplemented plants when compared to those in the Cd-alone- treated plants (Figure 5C).

### 2.5. Decline in Oxidative Damage in Response to 24-EBR Treatment

**Figure 6:**
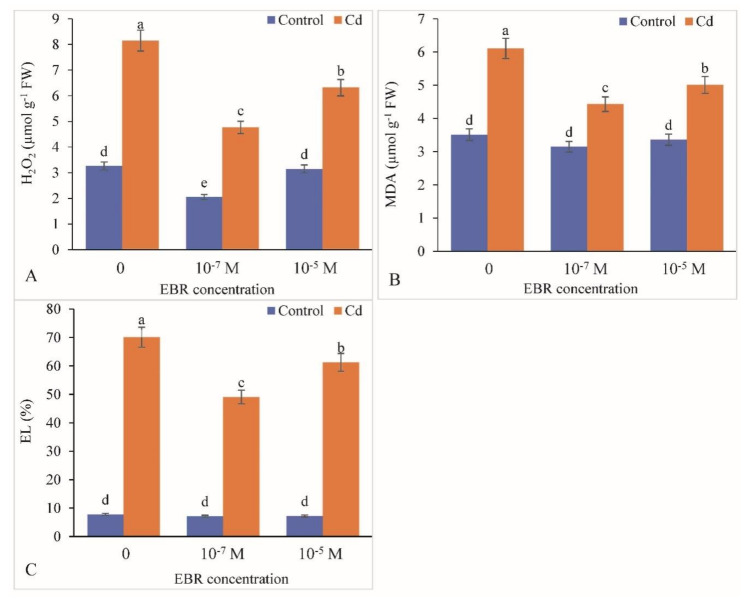
Supplementation of 24-Epibrassinolide regulates production of leaf (**A**) H_2_O_2_, (**B**) MDA and (**C**) EL in *B. juncea* under Cd (200 µM) stress. Values with different letters above the bars are significantly different at each measured time point (*p* < 0.05) (Mean ± S.E.).

Enhancement in production of H_2_O_2_ (150%) and MDA content (74.07%) under Cd stress was recorded with respect to the control plants. A decline of 41.47% and 22.45% in case of H_2_O_2_ levels and 27.49% and 18.0% in case of MDA content was recorded with 10^−7^ and 10^−5^ M treatments of 24-EBR, respectively, relative to the controls (Figure 6A,B). The electrolyte leakage showed 804% enhancement in Cd-stressed mustard leaves over the control. The 24-EBR foliar application lowered the electrolyte leakage by 30.02% and 12.58%, with 10^−7^ and 10^−5^ M EBR treatment, respectively, compared with the controls (Figure 6C).

### 2.6. Change in Antioxidative Defense Components by the 24-EBR Treatment

**Figure 7:**
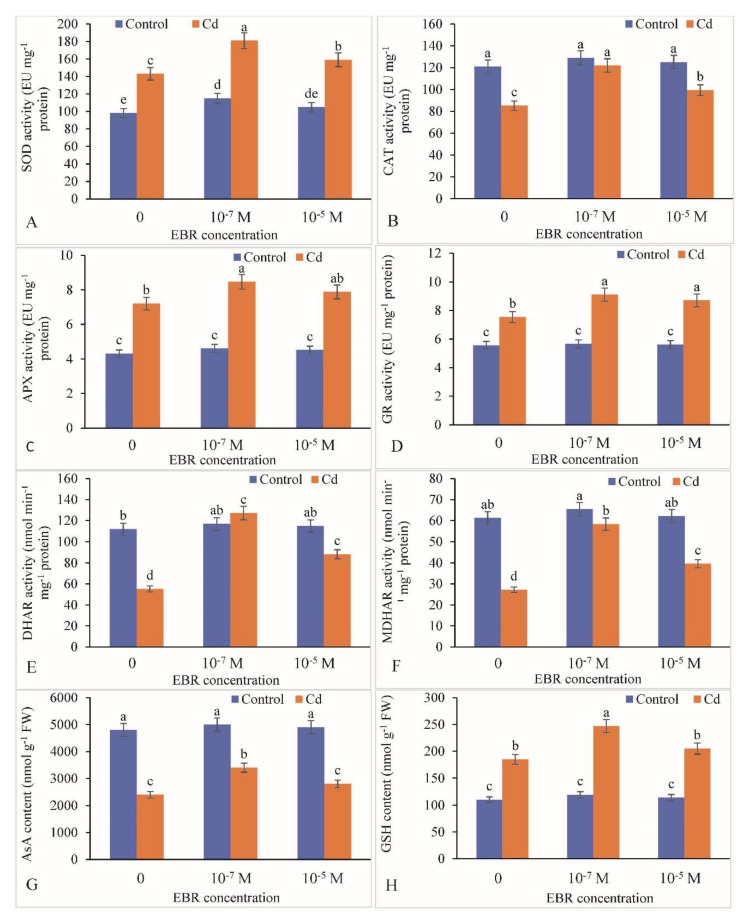
Supplementation of 24-Epibrassinolide enhanced the activity of leaf (**A**) SOD, (**B**) CAT, (**C**) APX, (**D**) GR, (**E**) DHAR, (**F**) MDHAR, (**G**) AsA content and (**H**) GSH content in *B. juncea* under Cd (200 µM) stress. Values with different letters above the bars are significantly different at each measured time point (*p* < 0.05) (Mean ± S.E.).

In comparison to the control plants, SOD activity was recorded to be enhanced by 45.59% in mustard leaves exposed to Cd stress. The SOD activity was elevated by 26.57% and 11.18% in 10^−7^ and 10^−5^ M EBR treatments, respectively, relative to that in the Cd-alone-stressed plants which revealed an increase of 45.59% (Figure 7A). Foliar application of 10^−7^ and 10^−5^ M 24-EBR also showed an enhancement in the activities of other enzymatic antioxidants including CAT (43.07% and 16.47%), APX (12.33% and 9.43%), GR (20.82% and 15.51%), DHAR (130.63% and 59.96%) and MDHAR (114.53% and 45.51%), respectively, relative to the respective controls (Figure 7B–F).

In contrast, the non-enzymatic antioxidants, i.e., AsA levels, decreased by 50.00% with Cd stress compared to the control. The supplementation of 24-EBR increased the AsA content by 41.66% at 10^−7^ and 16.66% at 10^−5^ M 24-EBR-supplemented plants over that in the Cd-alone-treated plants (Figure 7G). On the other hand, the GSH increased by 68.18% in Cd-stressed plants relative to the control. A further enhancement by 33.51% and 10.81% was observed with 10^−7^ and 10^−5^ M 24-EBR treatments in the Cd-stressed plants over that in the Cd-alone-treated plants (Figure 7H).

### 2.7. Methylglyoxalase System as Altered by 24-EBR Supplementation

**Figure 8:**
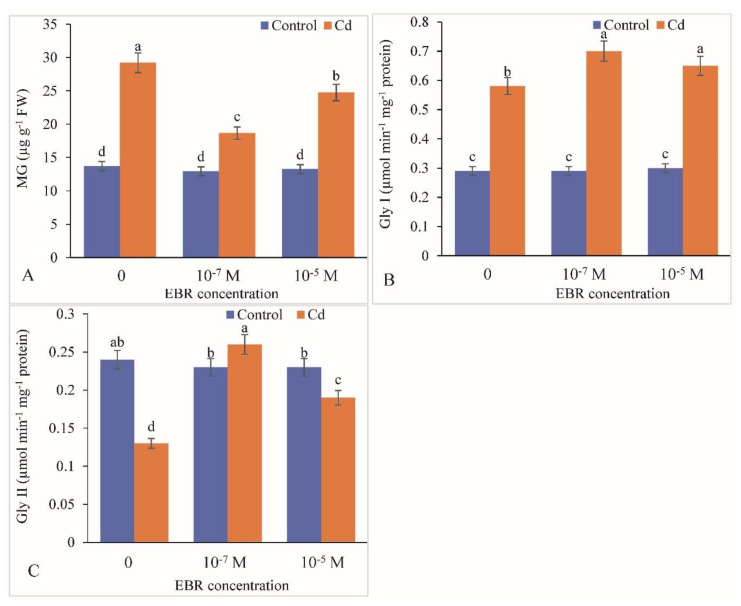
Supplementation of 24-Epibrassinolide declines leaf (**A**) MG content, but enhances activity of leaf (**B**) GlyI and (**C**) GlyII in *B. juncea* under Cd (200 µM) stress. Values with different letters above the bars are significantly different at each measured time point (*p* < 0.05) (Mean ± S.E.).

The Cd toxicity enhanced the MG level by 113.12% with reference to the control. Supplementation of 24-EBR decreased the MG content by 36.13% in 10^−7^ M and 15.29% in 10^−5^ M 24-EBR-treated plants relative to the controls (Figure 8A). The activity of GlyI was elevated by 100%, however, that of GlyII showed a decline by 45.83% in Cd-treated plants relative to the control. Supplementation of 24-EBR enhanced the GlyI and GlyII activities by 20.68% and 100% in 10^−7^ M and 12.06% and 46.15% in 10^−5^ M 24-EBR-supplemented plants, respectively, compared to those in the plants fed with Cd only (Figure 8B,C).

### 2.8. Cadmium Accumulation in Roots and Shoots

The Cd accumulation was comparatively more in roots (35.31 mg kg^−1^ DW) than that in shoots (22.42 mg kg^−1^ DW) in plants stressed with Cd alone. Supplementation of 24-EBR lowered the accumulation to 14.76 mg kg^−1^ DW (i.e., by 34.17%) and 24.74 mg kg^−1^ DW (i.e., by 29.93%) with 10^−5^ M EBR in shoot and root, respectively, relative to the controls (Table 1). Application of 10^−7^ M 24-EBR further lowered the Cd accumulation to 9.32 mg kg^−1^ DW (i.e., by 58.43%) in shoots and 18.45 mg kg^−1^ DW (i.e., by 47.75%) in roots compared with the controls (Table 1).

### 2.9. Translocation Factor and Shoot and Root Tolerance Indices as Altered by 24-EBR Application

The translocation factor decreased from 0.634 to 0.596 with 10^−5^ M EBR and from 0.634 to 0.505 with 10^−7^ M EBR application to the Cd-stressed plants (Table 1). The shoot tolerance index (STI%) increased from 55.81 to 86.12 with 10^−7^ M EBR and from 55.81 to 71.67 with 10^−5^ M 24-EBR. Similarly, root tolerance index (RTI%) also was enhanced from 60.65 to 79.51 and from 60.65 to 76.07 with 10^−7^ M EBR and 10^−5^ M EBR, respectively (Table 1).

### 2.10. Alterations in Yield Attributes

**Figure 9:**
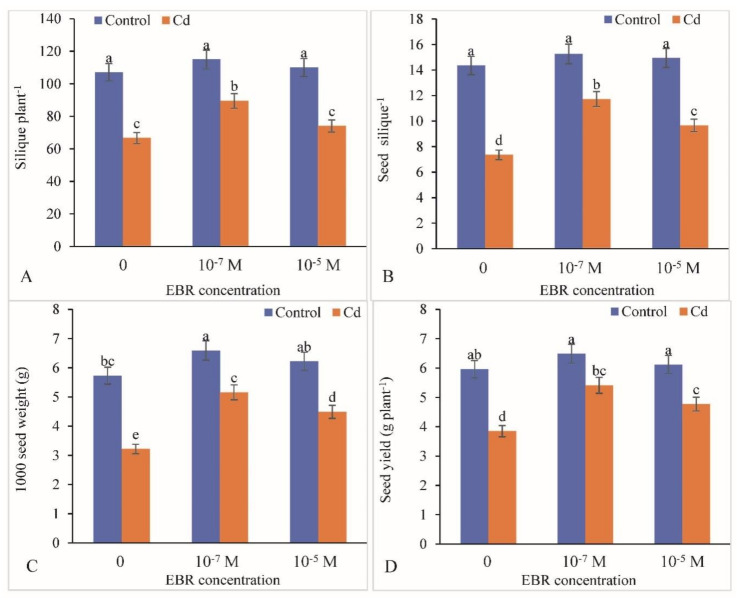
Foliar application of 24-Epibrassinolide enhanced the yield attributes of (**A**) siliques plant^−1^, (**B**) seed silique^−1^ (**C**) 1000 seed weight and (**D**) seed yield in *B. juncea* under Cd (200 µM) stress. Values with different letters above the bars are significantly different at each measured time point (*p* < 0.05) (Mean ± S.E.).

**Figure 10:**
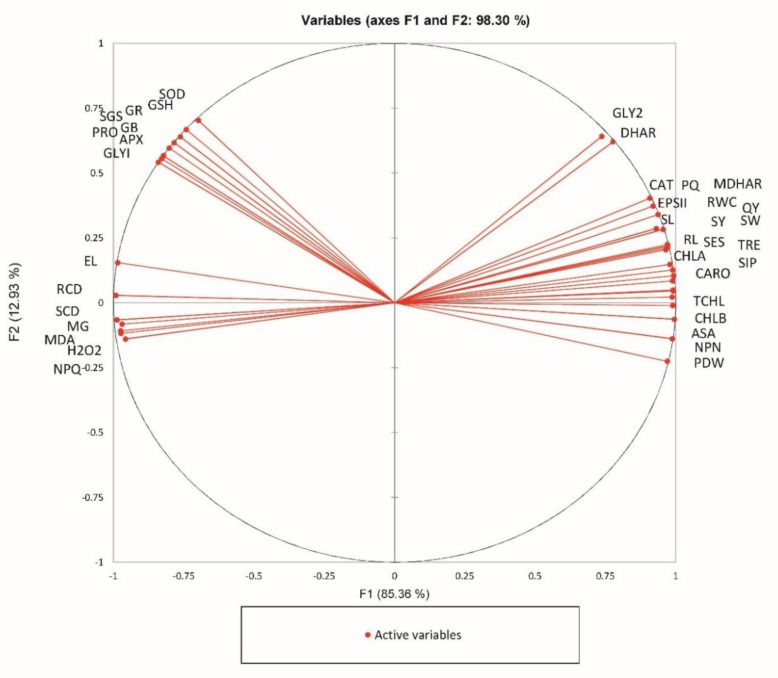
PCA analysis of foliar application of 24-Epibrassinolide to *B. juncea* under Cd (200 µM) stress. SL = Shoot Length; RL = Root Length; PDW = Plant Dry Weight; CHLA = Chl a; CHLB = Chl b; TCHL = Total Chl; CARO = Carotenoids; NPN = Net photosynthesis Pn; SGS = Stomatal conductance gs; TRE = Transpiration rate E; EPSII = Efficiency of PSII; QY = Quantum yield of PSII; PQ = Photochemical quenching; NPQ = Non-Photochemical quenching; SIP = Silique plant^−1^; SES = Seed Silique^−1^; SW = 1000 seed weight; SY = Seed yield; RWC = Relative water content; PRO = Proline; GB = Glycine betaine; EL = Electrolyte leakage; GLY2 = Gly II; SCD = Shoot Cd; RCD = Root Cd.

Under Cd stress, siliques per plant, seeds per silique, 1000 seed weight and seed yield decreased by 37.68%, 48.75%, 43.80% and 35.40%, respectively, compared with the respective controls (Figure 9A–D). The foliar application of 24-EBR enhanced the above-mentioned yield attributes to a significant level. The elevation in siliques per plant was by 11.15%, seeds per silique by 31.25%, 1000 seed weight by 8.55%, and seed yield by 23.89% in response to 10^−5^ M EBR compared with the respective controls. However, 10^−7^ M proved to be more beneficial in enhancing siliques per plant, seeds per silique, 1000 seed weight and seed yield, i.e., by 34.23%, 59.23%, 60.24% and 40.51%, respectively, compared with the controls (Figure 9A–D). Figure 10 reveals the correlations of different growth and yield parameters of mustard plants with various physio-biochemical attributes.

## 3. Discussion

Cadmium (Cd) is an undesired and non-nutritive metal that drastically disrupts the physiology of plants. The Cd-mediated inhibition of growth traits might be attributed to introversion of cell division and elongation in response to an irreparable damage caused to proton pumps by Cd ions. Furthermore, Cd also influences growth parameters as it adversely affects the rhizospheric habitat, root cells, elemental translocation, plant–water status, oxidative metabolism, and photosynthetic efficiency [34]. In the current experimentation, it was revealed that Cd treatment resulted in lowering the plant growth, dry mass, and seed yield. Corroborative reports regarding a decline in growth attributes in response to Cd stress has been reported in *Achnatherum inebrians* [35], *Mentha arvensis* [36] and *Triticum aestivum* [37]. Brassinosteroids (BRs) induced improvement in growth as well as yield of stressed crops, probably because of their imperative participation in cell division and elongation [38]. Moreover, the probable reason for this might be that BRs have been shown in earlier studies to regulate the expression of genes encoding xyloglucan endotransglucosylase, xyloglucan endotransglucohydrolase, cellulose synthase, and sucrose synthase, which are actively involved in promoting cell expansion and elongation [27]. Our findings agreed with the observations made by Jazi et al. [39] in rapeseed plants. The plant dry weight and seed yield were also enhanced by the 24-EBR application that could be attributed to BR-mediated stimulation in alteration in electrical properties of the biological membranes as suggested by Saeidnejad et al. [40]. Although both levels of BR used were effective in upregulating growth and metabolism, the lower level 10^−7^ µM of BR was found to be more effective than the higher one, i.e., 10^−5^ µM. Primarily, BRs stimulate growth by endorsing cell elongation, however, at later stages, the imperative role of BR in cell division was also affirmed. In recent years, it has been well elucidated that BRs modulate multifaceted aspects of growth and development in plants exposed to heavy metal stress, besides regulating cell division and elongation as well as other attributes including xylem differentiation, reproduction, photomorphogenesis and alleviation of heavy metal contamination and other environmental stresses [41].

Growth and development of plants is mainly modulated by photosynthesis in plants [42]. Inhibition in photosynthesis is generally initiated by degradation of chlorophyll by Cd [43]. The present results affirmed that the soils spiked with Cd result in a significant decline in chlorophyll and carotenoid contents. The reduction in the chlorophyll content might be a stimulatory mechanism adopted by plants, to induce increase in the activities of enzymes involved in the hydrolysis of a chlorophyll one such as chlorophyllase. Furthermore, it can be attributed to suppression in the activities of chlorophyll synthesizing enzymes such as δ-aminolevulinic acid dehydratase and protochlorophyllide reductase [44,45]. Similarly, the decline in carotenoid levels may result in disruption of PSII efficacy [46]. Corroborative observations of decline in photosynthetic pigment levels was observed in *Festuca arundinacea* plants exposed to Pb metal [47], and *Glycine max* plants subjected to Zn toxicity [48]. It was confirmed by the present observations that total chlorophyll, chl a, chl b, and carotenoid levels were replenished by the exogenous application of 24-EBR. Similar results of up-regulation in chlorophyll content were observed by Verma et al. [49] in groundnut plants in response to 24-EBR application. This might be attributed to BR’s involvement in augmenting photosynthetic competence of plants. Similarly, Kohli et al. [50] showed a comparable augmentation in chlorophyll concentration and attributed the specific response to decline in expression of *CHALASE* gene (chlorophyllase, a chlorophyll-degrading enzyme) due to 24-EBR application. The foliar application of 24-EBR considerably enhanced the biomass by augmenting CO_2_ assimilation capacity, Fv/Fm ratio and photosynthetic pigment levels in Cd-stressed plants [51]. Imaging of tobacco leaf mesophyll cells captured by employing transmission electron revealed disruption of cell wall and membranes due to Cr stress. Although, the treatment with 24-EBR resulted in enhancing protection of chloroplast and maintaining the ultrastructural components of grana and thylakoids [51]. Retardation in the gas exchange attributes *viz.* net photosynthetic rate and transpiration rate was recorded in the current study. A similar information of down-regulation in the gas exchange attributes was earlier reported by Khan et al. [52] who revealed reduction in cellular CO_2_, stomatal conductance, net photosynthetic rate, and transpiration rate in *Solanum lycopersicum* plants exposed to abiotically strained conditions. This might have been possibly due to the deleterious impact of a metal on photosynthetic pigments, which may eventually result in lowering the light enthralling capacity of the photosystems [53]. A similar decline in gas exchange attributes has been revealed in *Triticum aestivum* plants under Cd stress [54], *Zea mays* plants under Cd and As stress [55], and *Glycine max* plants under Cd stress [56]. The current study affirmed that the supplementation of 24-EBR resulted in elevation of all gas exchange attributes. A similar observation of elevation in gas exchange attributes was reported by Xia et al. [57] in cucumber plants due to BR’s application. Enhancement in gas exchange parameters such as photosynthetic rate by 29% and stomatal conductance by 18% with the 24-EBR treatment was reported by Shahid et al. [58] in salt-stressed pea plants. Similarly, other photosynthetic attributes including efficiency of PSII, quantum yield of PSII and photochemical quenching were augmented in response to 24-EBR foliar application. This might have been possibly due to the active participation of BRs in numerous light- and hormone-modulated photosynthetic metabolisms [59,60].

Under Cd toxicity, RWC was reduced significantly. Our annotations can be accorded with the findings of Azhar et al. [61] who suggested an imbalance in water status, i.e., an elevation in the water content of *Helianthus annuus* plants exposed to heavy metal. It might have been due to enhancement in water content in response to metal toxicity as a consequence of stomatal closure, which eventually may result in compromised atmospheric carbon-fixation [62]. Further elevation in RWC was observed in the present study. On the other hand, the electrolyte leakage was elevated in response to Cd stress and lowered in response to 24-EBR possibly due to BR’s stimulated alteration in electrical properties of biological membranes as suggested by Saeidnejad et al. [40]. The levels of osmolytes increased in response to Cd treatment as well as 24-EBR treatment. The exposure of heavy metals results in stimulation of synthesis of osmoregulators such as proline, sugars, and GB. These osmolytes may have an imperative role in providing protection against metal toxicity by modulating cellular osmotic status, stabilization of proteins and enzyme activities, and detoxification of free radical species as well as maintenance of redox homeostasis [63]. 24-EBR application resulted in a further enhanced accumulation of osmolytes, specifically that of proline as earlier reported by Anuradha and Rao [64] in *Raphanus sativus*, and Irfan et al. [65] in *B. juncea* plants exposed to metal toxicity. The stimulation of osmolyte levels in response to BR’s treatment has a latent role in maintenance of the cellular metabolism [66].

ROS production is induced in response to Cd toxicity in plants and is a well-recognized mechanism in plants, as Cd is a non-redox transition metal that stimulates over-synthesis of ROS indirectly by suppressing the activity of antioxidative defense response [67]. It also results in enhancement in the activity of NADPH oxidases [68]. ROS overproduction induces lipid peroxidation of the biological membranes thereby resulting in large amounts of MDA. A similar elevation in ROS levels *viz.* H_2_O_2_ and MDA was observed in the present investigation in response to Cd stress. Similar observations showing rise in ROS levels have been recorded in *Achnatherum inebrians* and *Mentha arvensis* plants exposed to Cd stress [36,47]. The levels of ROS were lowered by the 24-EBR treatment in the current study; these observations were in line with the earlier findings of Bhardwaj et al. [69] in *Zea mays*, Choudhary et al. [70] in *Raphanus sativus*, and Kohli et al. [50] in *Brassica juncea* plants exposed to metal stress and supplied with 24-EBR. The decline in the levels of ROS by 24-EBR application is attributed to the activation of both enzymatic and non-enzymatic components of the antioxidative defense system [71]. The activities of chief antioxidants as well as detoxification enzymes are relatively up-regulated and so are the protein and transcript levels [32]. The activity of antioxidative enzymes including SOD, CAT, APX, GR, DHAR and MDHAR were elevated in response to Cd treatment and exogenous supplementation of 24-EBR in the present study. The possible reason for elevation in enzyme activities in response to 24-EBR treatment could be due to the potential of BRs to modulate the expression of various genes that mediate the stimulation and deactivation as well as de *novo* synthesis of various antioxidative enzymes [72]. An additional probable rationale for augmented activities of antioxidative enzymes could be attributed to BR signaling kinase 1 (BSK 1) which positively modulates the endogenous levels of salicylic acid, by eventually triggering the alleviation of the adverse impact of the oxidative burst [73]. Application of 24-EBR elevated the tolerance of tomato plants exposed to ZnO (zinc oxide) nanomaterial-induced stress by augmenting the activity of antioxidative enzymes and improving the redox status [74]. Comparable reports of elevation in the gene expression of *SOD*, *POD*, *CAT*, *GR*, *DHAR* and *GST* genes might have been the reason for enhanced activity of antioxidative defense system in response to 24-EBR treatment [50]. Cadmium-stressed plants showed elevated levels of MG, and the activities of Gly I and Gly II, however, supplementation with 24-EBR further enhanced their levels/activities. In addition to the antioxidative defense cascade, plants have developed other strategies to facilitate the detoxification of methyglyoxal, i.e., by activation of the glyoxalase system (Gly I and Gly II) [18,75]. The enhancement in the amount of MG might be toxic to plants and can cause a subsequent depletion of GSH. In another study, it has been reported that exogenous application of 24-EBR in pea plants resulted in enhancement in sequestration of Gly I and Gly II and has been affirmed to defend the plants against Cd stress-induced augmentation in MG accumulation [76]. Furthermore, it was suggested by Jan et al. [63] that 24-EBR might lead to elevation in intake of essential elements such as Ca and in situ levels of certain hormones which are involved in safeguarding of the glyoxalase pool as well as by lowering the toxicity of MG in response to Cd treatment. They further added that detoxification of MG is not sufficient to alleviate toxic symptoms of Cd stress, and maintenance of Gly I and Gly II activities both assist GSH refurbishment.

Cadmium accumulation was shown to be comparatively more in the roots than in the shoots in the present piece of work. The possible reason for this could be due to higher mobility of Cd in soil and plants, which may result in its easy absorption by plant roots [48]. Supplementation of 24-EBR lowered the accumulation with 10^−5^ M and 10^−7^ M 24-EBR in both roots and shoots. Application of 24-EBR affirmed lowered Cd accumulation in response to enhancement in Ca absorption in order to maintain the ionic balance in the plant system [77,78]. Moreover, 24-EBR has been widely reported to elevate the uptake of various cations including K^+^, Ca^2+^ and Mg^2+^ in the root system and these cations are preferably translocated to the young tissues present in the leaves via stellar bundles, consequently lowering the Cd uptake [63].

The translocation factor of transport of Cd ions was lowered by 24-EBR supplementation and there was a subsequent elevation in the shoot and root tolerance indices. 24-EBR application is suggested to have caused an elevation in inclusion of crucial inorganic ions, and decreased intake of noxious metal ions, which consequently uphold the ionic stability of the plant system. These essential ions include K^+^, Na^+^, Ca^2+^ and Mg^2+^ in younger tissues and Mg^2+^/Na^+^ and Ca^2+^/Na^+^ in the roots and K^+^/Na^+^ in the petioles [79,80]. Taken together, exogenous application of 24-EBR augmented the tolerance to Cd stress which could be ascribed to considerable improvement in the levels of photosynthetic pigments, gas exchange attributes, antioxidant defense signaling and ROS scavenging as well as osmoregulation [81,82].

The PCA diagram (Figure 10) shows analyzed correlated parameters. The close variables in the diagram are significantly positively correlated. There are four major correlated groups: group I. GLY2 and DHAR; group II. CAT, PQ, EPS II, SL, RL, MDHAR, RWC, QY, SY, SW, SES, TRE, CHLA, SIP, CARO, TCHL, CHLB, ASA, NPN, and PDW; group III. SOD, GSH. GR, GB, APX, SGS, PRO, and GLYI; and group IV. EL, RCD, SCD, MG, MDA, H2O2, and NPQ. The group I and III, group II and IV are on the opposite side of the center, so they are negatively correlated.

## 4. Materials and Methods

The viable seeds of brown mustard (*Brassica juncea* (L.) Czern.) were surface-sterilized with 0.1% sodium hypochlorite and then washed properly with distilled water for 5 min. After sterilization, the seeds were sown in pots containing sand, perlite and peat (1:1:1). After 4 days of germination, the seedlings were provided with 200 mL Hoagland solution (full strength) to each pot daily for 10 days. After 10 days, the plants were exposed to cadmium (0 and 200 µM CdSO_4_) dissolved in Hoagland solution for 10 days. The control plants received Hoagland solution only. The control and Cd-treated plants were supplemented through foliage with 10^−7^ and 10^−5^M 24-EBR (20 mL per pot). For this purpose, Teepol (0.1%) as a surfactant was mixed in the EBR solution. The plants were harvested after 45 days of sowing, followed by performing independent experiments (each with three replicates of each treatment) in order to analyze the results statistically. The plants were grown up to day 45 under controlled conditions in a greenhouse with temperature 25/15 day/night, 70–75% relative humidity and 18 h of light period. After 45 days of growth, the plants were harvested and fresh leaves were used for the analyses of different biochemical parameters.

### 4.1. Determination of Growth Traits

A manual scale was used for the measurement of lengths of roots and shoots. Known samples were kept for drying in an oven at 70 °C, and after 72 h, the dry weight of the samples was measured.

### 4.2. Determination of Pigment Content

Fresh leaf sample (each 200 mg) was homogenized in 0.2 mL of 80% acetone and then centrifuged at 12,000× g for 5 min. The supernatant collected and their absorbance at 663 and 645 nm for the assessment of chlorophyll content and at 480 and 510 for the estimation of carotenoid content was measured by employing a spectrophotometer [83].

### 4.3. Estimation of Cd

For the estimation of Cd, dried plant samples (0.5 g) were digested in an acid mixture and Cd levels in the digested leaf samples were measured with an atomic absorption spectrophotometer (Perkin-Elmer Analyst Model 300) and the values presented as µmol g^−1^ DW.

### 4.4. Cadmium Translocation Factor and Tolerance Index

The translocation factor for Cd was worked out as the quotient of Cd content in shoot by comparing it with that of the root. The shoot and root tolerance indices were calculated by the following formulae:

(1) $$STI\;\left(\%\right)=\frac{Shoot\;length\;in\;Cd\;treated\;plants}{Shoot\;length\;in\;control\;plants}\;\times 100$$

(2) $$RTI\;\left(\%\right)=\frac{Root\;length\;in\;Cd\;treated\;plants}{Root\;length\;in\;control\;plants}\;\times 100$$

### 4.5. Gas Exchange Attributes

Gas exchange attributes including net photosynthetic rate (*Pn*), transpiration rate (*E*) and stomatal conductance (*g_s_*) were estimated using an IRGA (LCA-4 model Analytical Development Company, Hoddesdon, England) using fully expanded leaves between 10:00 h and 12:00 h in full and bright sunlight.

### 4.6. Estimation of Chlorophyll Fluorescence

A PAM chlorophyll fluorimeter (H. Walz, Effeltrich, Germany) was used for the determination of chlorophyll fluorescence parameters. The parameters were estimated in a fully stretched leaf employing the method proposed by Li et al. [84].

### 4.7. Determination of Leaf Relative Water Content (LRWC)

LRWC was analyzed by employing the method of Yamasaki and Dillenburg [85]. The fresh weight of the leaves was immediately noted after harvesting. To obtain turgid weight of the leaves, they were dipped in distilled water in a Petri dish, which was covered. During the entire period of imbibition, the leaves are weighed at intervals, only after wiping water off the leaf surface with the help of a filter paper. The Petri dishes were kept under a dim light, 20 µmol m^−2^ s^−1^ intensity and exposed to natural temperature. Following formula was used for the calculation of LRWC:

LRWC = Fresh weight − Dry weight/Turgid weight − Dry weight(3)

### 4.8. Glycine Betaine and Proline Estimation

Leaf tissue (each 300 mg) was triturated in sulfosalicylic acid and after centrifugation at 10,000× *g* for 10 min, equal volumes of the supernatant (2 mL each), acid ninhydrin and glacial acetic acid were mixed. After incubation for 1 h at 100 °C, the mixture was kept in an ice bath. Toluene was used for the extraction of proline. Optical density was taken at 520 nm and proline content presented as µg g^−1^ FW [86]. 

Glycine betaine (GB) was estimated following Grieve and Grattan [87]. A proportion measuring 0.5 g of dried plant leaves was ground in 5 mL of distilled water/toluene mixture and was kept as such for 24 h and then filtered. To 0.5 mL of the prepared extract, 2N HCL and 0.1 mL PI were added and incubated in an ice bath for 1.5 h with constant shaking. To the extract, further 2 mL of ice cold distilled water and 10 mL of 1, 2-dichloroethane were added. The OD of the lower aqueous layer was recorded at 365 nm and the GB expressed as µg g^−1^ FW. GB levels were determined by a standard betaine hydrochloride curve.

### 4.9. Determination of H_2_O_2_, MDA and EL

Each leaf sample (200 mg) was extracted in 0.1% TCA and the mixture was centrifuged for 10 min at 10,000× *g*. To the supernatant (0.5 mL), 100 mM potassium phosphate buffer (pH 7.0, 0.5 mL) was mixed followed by addition of 1 M potassium iodide. Absorbance was noted at 390 nm and H_2_O_2_ was presented as nmol g^−1^ FW [88]. 

The method described by Madhava Rao and Sresty [89] was adopted for the estimation of malondialdehyde (MDA) content by reacting a known quantity of the extract with thiobarbituric acid (prepared in 20% TCA) at 100 °C for 30 min. The samples were kept in an ice bath and after cooling, the samples were centrifuged for 10 min at 10,000× *g*. The OD was recorded at 532 and 600 nm.

For the appraisal of electrolyte leakage, 20 leaf discs of uniform size were placed in a test tube containing appropriate amount of DW and its electrical conductivity (EC0) was measured. Then the leaf discs were boiled at 50 °C for 20 min and EC (EC1) was again measured. After that, the same sample was boiled at 100 °C for 10 min and EC (EC2) was measured again [90]. EL was calculated using the following formula:

EL = EC1 − EC0/EC2 − EC0 × 100(4)

### 4.10. Appraisal of Activities of Antioxidative Enzymes and Components of the Ascorbate-Glutathione Cycle

For extraction of antioxidant enzymes, fresh sample of leaves (each 0.5 g) were crushed in chilled potassium phosphate buffer (pH 7.0, 100 mM) and polyvinyl pyrrolidone solution (1%). The crushed material was subjected to centrifugation at 12,000× *g* at 4 °C for 30 min. The collected supernatant was utilized for the analysis of various enzymes.

***Superoxide dismutase (SOD*, *EC1.15.1.1) activity:*** The activity of SOD was calculated by examining the reduction of NBT (nitroblue tetrazolium) photochemically [91]. The OD was noted at 560 nm and SOD was determined as EU mg^−1^ protein.

***Catalase (CAT*, *EC1.11.1.6) activity*:** CAT activity was appraised following Aebi [92] and decline in H_2_O_2_ was noted for 2 min at 240 nm. The reaction mixture comprised of potassium phosphate buffer (50 mM, pH-7) and 15 mM H_2_O_2_. To the reaction mixture, plant extract was added and absorbance was recorded at 240 nm for 2 min. CAT activity was determined as EU mg^−1^ protein.

***Ascorbate peroxidase (APX*, *EC1.11.1.1) activity:*** For the estimation of APOX activity, 2.5 mL of the reaction mixture comprised phosphate buffer (1940 µL), 250 µL of ascorbic acid, 250 µL of hydrogen peroxide and plant extract (60 µL). APX activity was analyzed by scrutinizing the H_2_O_2_-dependent oxidation of ascorbate. The decline in absorbance was recorded at 290 nm for 2 min [93]. APX was determined as EU mg^−1^ protein.

***Glutathione reductase (GR*, *EC1.6.4.2) activity:*** GR activity was evaluated by the method given by Foster and Hess [94]. The OD was recorded for 3 min at 340 nm and the GR activity was determined as EU mg^−1^ protein.

***Monodehydroascorbate reductase (MDHAR*, *EC1.6.5.4) activity*:** The protocol described by Miyake and Asada [95] was utilized for the evaluating of MDHAR activity. The OD was noted at 340 nm and the activity was measured as NADPH oxidized (EU mg^−1^ protein).

***Dehydroascorbate reductase (DHAR*, *EC1.8.5.1) activity*:** The Nakano and Asada [93] method was adopted for the estimation of DHAR activity and the OD was noted at 265 nm. The DHAR activity was determined as EU mg^−1^ protein.

***Ascorbic acid (AsA) and glutathione:*** The methods described by Huang et al. [96] and Yu et al. [97] were employed for the estimation of contents of ascorbate and glutathione, respectively.

### 4.11. Estimation of MG, Gly I (EC4.4.1.5) and GlyII (EC3.1.2.6)

For the estimation of MG content, the Wild et al. [98] protocol was employed and the OD was recorded at 288 nm. The levels of MG were appraised by employing a standard curve.

The protocols outlined by Hossain et al. [76] and Mostofa and Fujita [99] were employed for the estimation of Gly 1 and Gly II activities. The OD was recorded at 240 nm and the Gly 1 and Gly II activities were presented as µmol min^−1^ mg^−1^ protein.

### 4.12. Yield Attributes

At maturity, the plants were harvested and the yield-related data were recorded.

### 4.13. Statistical Analysis

The experimental data were analyzed with statistical software package STATISTIX 10 using one-way analysis of variance (ANOVA) and Bonferroni Multiple group comparison. Figures were produced using Microsoft Excel. The correlation circle was done using Principal Component Analysis (PCA) method in XLstat software.

## 5. Conclusions

The current study establishes that seed treatment/priming with 24-Epibrassinolide augmented growth and photosynthetic efficiency, and lowered the ROS levels of Cd-stressed *Brassica juncea* plants by positively modulating the antioxidative defense system and regulating the levels of osmolytes. 24-Epibrassinolide application also replenished the contents of antioxidants viz. glutathione and ascorbic acid. It is evident from the study that 24-Epibrassinolide can be effectively employed to alleviate the adverse effects of Cd on *B. juncea* plants. The supplementation with phytohormones to metal-contaminated soils can be a sustainable strategy to enhance crop productivity and health. Hormone treatment resulted in improved photosynthetic pigments possibly by mediating enhancement in expression of key enzymes involved in the synthesis of pigments, and subsequently elevated the efficiency of gaseous exchange attributes. The application of 24-Epibrassinolide is widely reported to be involved in regulating the ROS optimal concentrations and modulating the functioning of plants under stressful conditions by augmenting the activities of non-enzymatic and enzymatic antioxidants. Reduced ROS levels and membrane damage aid in uptake and accumulation of essential micronutrients. 24-Epibrassinolide have an imperative role in maintaining the levels of osmoregulators including proline and GB which are involved in the osmotic system of the plants as osmolytes are contemplated as vital for various processes in plants. 24-Epibrassinolide caused the recovery of various metabolites viz. MG, GLY I and GLY II, revealing their potential role in reducing the uptake of Cd which eventually resulted in reducing the oxidative stress. Brassinosteroids, therefore, via causing alterations in various physiological processes can offset the damaging effects of Cd toxicity and protect plants from metal stress.

We can foresee that 24-Epibrassinolide supplementation has a potential for promoting growth, photosynthetic efficacy and stimulating antioxidative potential in most plants subjected to metal stress. It is now widely reported that different priming agents trigger specific genes in seeds, which could regulate some specific metabolic processes that remain functional throughout the plant life cycle in plants subjected to stressful cues. Thus, future research should focus on the identification of genes as well as proteins involved in a number of key physio-biochemical processes in a primed seed such as water uptake, metabolisms of carbohydrates, proteins and fat, mobilization of reserves, regulation of cell cycle, etc.

## Figures and Tables

**Table 1:** Supplementation of 24-Epibrassinolide regulates Cd accumulation in shoot and root, decreases translocation factor and enhances shoot and root tolerance index in *B. juncea* under Cd (200 µM) stress (Mean ± S.E.).

EBR (Molar)	Shoot Cd (mg kg^−1^ DW)		Root Cd (mg kg^−1^ DW)		Translocation Factor	Shoot Tolerance Index (STI%)	Root Tolerance Index (RTI%)
	Control	Cd	Control	Cd			
0	ND	22.42 ± 0.595 ^a^	ND	35.31 ± 0.917 ^a^	0.634 ± 0.029 ^a^	55.81 ± 5.05 ^c^	60.65 ± 5.62 ^c^
10^−^^7^	ND	9.32 ± 0.327 ^c^	ND	18.45 ± 0.461 ^c^	0.505 ± 0.013 ^c^	86.02 ± 7.33 ^a^	79.51 ± 7.10 ^a^
10^−^^5^	ND	14.76 ± 0.326 ^b^	ND	24.74 ± 0.529 ^b^	0.596 ± 0.021 ^b^	71.67 ± 6.85 ^b^	76.07 ± 6.95 ^b^

Values with different letters are significantly different at each measured time point (*p* < 0.05). ND = not detected.
